# The value of sonication on orthopaedic implants in an everyday clinical setting – an exploratory study

**DOI:** 10.1186/s12891-023-06796-x

**Published:** 2023-08-29

**Authors:** Diana Salomi Ponraj, Thomas Falstie-Jensen, Holger Brüggemann, Jeppe Lange

**Affiliations:** 1 Department of Clinical Medicine, Aarhus University, Aarhus, 8000 Denmark; 2 Department of Orthopaedic Surgery, Aarhus University Hospital 8200, Aarhus, Denmark; 3 Department of Biomedicine, Aarhus University, Aarhus, 8000 Denmark; 4 Department of Orthopaedic Surgery, Regional Hospital, Horsens, 8700 Denmark

**Keywords:** Sonication, Orthopaedic implant infections, *Cutibacterium*

## Abstract

**Background:**

Sonication of removed orthopaedic implants in suspected implant-associated infections (IAI) is widely applied internationally. However, evaluation of the utility of sonication on all implants removed in everyday standard practice is scarce. This exploratory study was performed to evaluate the application of sonication fluid (SF) culture on removed orthopaedic implants, irrespective of the reason for removal.

**Methods:**

Out of 100 removed orthopaedic implants collected between August 2019 and September 2020, 77 implants with availability of concurrent tissue culture samples were included in the study. Removed implants were categorized into a confirmed or suspected IAI group and a presumed aseptic group based on pre-operative diagnosis by the responsible surgeon. Implants were sonicated and SF culture performed under both aerobic and anaerobic conditions. The significance of all bacterial isolates was evaluated based on the CFU/mL cut-offs of the EBJIS guidelines, except for *C. acnes* where additional investigations were performed.

**Results:**

The results of SF culture in the two groups were compared with their corresponding tissue cultures. Out of the 12 cases in the confirmed/suspected IAI group, SF culture was positive in 11 cases and had increased diagnostic yield in two (17%) cases compared to tissue culture. Increased diagnostic yield of SF compared to tissue culture was seen in seven (11%) of the 65 implants in the presumed aseptic group. If growth of *Cutibacterium* species isolates were interpreted based on EBJIS cut-off for SF culture instead of the study-specific criteria, then two isolates considered to represent infection might have been missed while three other isolates considered contaminants would have fallen under the ‘infection confirmed’ category in the EBJIS guidelines.

**Conclusion:**

Sonication with SF culture has increased diagnostic yield compared to tissue cultures in all implants irrespective of reason for removal. However, positive SF cultures with *Cutibacterium* species should always be interpreted with extreme care.

## Introduction

Orthopaedic implant-associated infections (IAI) can result in the need for repeated surgeries, extended hospitalization, prolonged antibiotic usage, and poor clinical outcome [1, 2]. The causative agents of IAI are varied and include both highly virulent bacteria like *Staphylococcus aureus* with a well-recognized role in IAI, as well as slow-growing, Gram positive anaerobic bacteria (SGAB) like *Cutibacterium acnes* whose role is more controversial, especially when isolated from presumed aseptic cases [1, 3–10].

Culture of tissue samples obtained from the peri-implant area are traditionally used to detect the causative agents in suspected IAI [11]. However, bacteria causing IAI tend to form biofilms on the surface of implants and can be missed by standard tissue cultures [12, 13]. This may be alleviated by sonication, where biofilms are dislodged into the sonication fluid (SF), which can then be cultured [12–15]. However, sonication may not be routinely performed during removal of orthopaedic implants in many institutions, especially in non-joint prosthesis hardware, unless an infection is readily suspected by the responsible surgeon. Previous studies have shown that the refrained use of SF cultures might lead to misdiagnosing an implant failure as aseptic, especially in cases with low-grade IAI without overt features of infection [7]. However, the value of utilizing SF culture in all clinically aseptic or apparent mechanical failure cases, is currently unclear [7].

In 2019 we initiated a SGAB project with a focus on sonication and optimized anaerobic growth conditions to explore the prevalence and nature of SGAB in orthopaedic implants. We have previously reported on data obtained in this project with a singular focus on *C. acnes* [16]. The purpose of this study, which is based on all implants acquired in the previous study, is to evaluate the application of sonication with SF culture in addition to tissue cultures on removed orthopaedic implants, irrespective of the type of implant or reason for removal.

## Methods

The study was performed as an exploratory cross-sectional study, based on a research database, with prospective data collection. The implants were collected and processed as described previously [16]. In brief, one hundred orthopaedic implants that were removed between August 2019 and September 2020, irrespective of the type of implant or reason for removal, were collected from the Department of Orthopaedic Surgery, Aarhus University Hospital and transported to the Department of Biomedicine, Aarhus University, Denmark. Implants (n = 33) where tissue samples were not obtained were excluded in the present study leaving a total of 77 implants for evaluation.

### Tissue culture

At the discretion of the operating surgeon, tissue samples were obtained for routine culture during implant removal, as described by Kamme and Lindberg [17]. The tissue samples were processed according to standard procedures at the local Department of Clinical Microbiology. The results of tissue sample cultures, as well as relevant implant related data were obtained from the patients’ electronic medical records. Five tissue biopsies were taken in all cases except for four (5%), where less than five biopsies were taken. No bacterial growth was seen in any of these four cases (both in SF and tissue cultures). We defined isolates in tissue cultures as potential contaminants, if a bacterial species was isolated from only one of five tissue biopsies [17].

### Sonication fluid culture

All implants were sonicated using a standardized method of 30 s vortexing, sonication for one minute at 100% followed by further 30 s of vortexing [18]. The SF obtained was inoculated on three different agar media (blood agar, fastidious anaerobic agar and reinforced clostridial agar). A total of ten plates per implant were used, with nine plates for anaerobic incubation and one plate for aerobic incubation. This methodology was chosen due to the context of the SGAB project. For aerobic incubation, uncentrifuged SF was used while for anaerobic incubation, both uncentrifuged (three plates) and centrifuged SF (six plates) were inoculated. Incubation was performed up to three days for the aerobic cultivation and 28 days for anaerobic cultivation. All bacterial growth was quantified using colony forming units/ml (CFU/ml) and bacteria were identified with 16 S rRNA gene sequencing [16].

The significance of *C. acnes* isolates from SF was determined in an in-depth evaluation in the SGAB project where a combined analyses of culture-dependent and -independent methods were used [16]. The culture-dependent methods included single-locus sequence typing (SLST) and whole genome sequencing of all *C. acnes* isolates obtained from culture [16]. In the culture-independent SLST amplicon-based next-generation sequencing of SF, the various *C. acnes* SLST types present in the SF were detected directly from the SF and their relative abundancies measured [16]. In instances of discrepancies between the two methods, especially when the *C. acnes* SLST types isolated from culture were not detected by the culture-independent methods, the *C. acnes* isolates were considered contaminants [16]. In all other isolates, European Bone Joint Infection Society (EBJIS) criteria for SF culture, defined by bacterial growth ≤ 50 CFU/ml in uncentrifuged SF or ≤ 200 CFU/ml in centrifuged SF was used to identify likely contaminants [19].

The study was registered with Region Midtjylland with reference number 661,624 and carried out in accordance with relevant guidelines and regulations. The need for ethical approval was waived by Central Denmark Region ethical committee. Informed consent was obtained from all patients before their participation in the study. All data reported in the study was prospectively entered into our SGAB project at Aarhus University REDCap system [20], and all patient identifiers were removed before downloading the data for analysis. Data are presented as proportions in percentages.

## Results

Out of the 77 implants included in the study, majority (n = 69; 90%) were arthroplasties. Shoulder (n = 34; 44%) was the most common anatomic location, followed by the hip (n = 18; 23%) and knee (n = 17; 22%). Twelve (16%) of the 77 implants included in the study were removed due to infection as noted by the responsible surgeon in the patient chart. The remaining 65 (84%) implants were removed for presumed non-infectious reasons, including mechanical failure and pain.

Bacteria were isolated from SF culture in 35 (46%) of the 77 implants. The reason for implant removal was infection in 11 (31%) of the 35 cases.

Positive tissue cultures were noted in 18 (23%) of the 77 cases. In 10 (56%) of the 18 cases with positive tissue culture the surgeon had registered infection as the reason for removal of the implant.

Data from the 12 (16%) cases in which the implant was removed due to infection are depicted in Table 1. There was concordance between SF and tissue culture results in 11 (92%) cases. In the remaining case (No.2), in which a sinus tract was present, *C. acnes* was isolated only from SF culture, with a low CFU/mL. Noticeably, in one case, *Bacteroides fragilis* was isolated from only one of five tissue specimens, while in SF culture it was isolated with > 250 CFU/ml.

**Table 1:** Details of the twelve implants in the confirmed/suspected IAI group

No.	**Joint**	**Type of Implant**	Tissue culture^a^	**Sonication fluid culture**		
				**Organism**	**Colony forming units/ml** ^**c**^	**Time to growth in days**
1	Shoulder	Arthroplasty	*C. acnes* (4/5)	*C. acnes* ^*b*^	100	7
				*C. namnetense* ^*d*^	40	7
2	Shoulder^e^	Plates/screws	No growth (0/5)	*C. acnes* ^*b*^	10	3
3	Hip	Arthroplasty	*S. aureus* (4/5)	*S. aureus*	> 250	1
4	Elbow	Arthroplasty	*S. aureus* (3/5)	*S. aureus*	> 250	1
5	Hip	Arthroplasty	*S. aureus* (5/5)	*S. aureus*	> 250	1
6	Shoulder	Arthroplasty	*S. epidermidis* (5/5)	*S. epidermidis*	> 250	2
7	Hip	Arthroplasty	*S. mutans* (3/5)	*S. mutans*	> 250	2
8	Knee	Arthroplasty	*S mitis* (5/5)	*S. mitis*	> 250	1
9	Shoulder	Arthroplasty	*P. aeruginosa* (5/5)	*P. aeruginosa*	> 250	1
10	Knee	Arthroplasty	*F. magna* (3/5)	*F. magna*	> 250	4
11	Knee	Arthroplasty	*B. fragilis* (1/5)	*B. fragilis*	> 250	3
12	Shoulder^e^	Arthroplasty	No growth (0/5)	No growth	-	-

^a^ Numbers in parentheses after the bacterial species name give the number of cultures that were positive for the bacteria/total number of cultures taken

^b^ Significance of *C. acnes* isolates were based on our previous publication.

^c^ The CFU was calculated based on growth in uncentrifuged sonication fluid.

^d^ Classified as contaminant

^e^ Clinical diagnosis of IAI was based on the presence of sinus tract

*C. acnes* – *Cutibacterium acnes*, *C. namnetense – Cutibacterium namnetense*, *S. aureus* – *Staphylococcus aureus*, *S. epidermidis* – *Staphylococcus epidermidis*, *S. mutans* – *Streptococcus mutans*, *S. mitis* – *Streptococcus mitis*, *P. aeruginosa* – *Pseudomonas aeruginosa*, *F. magna* – *Finegoldia magna*, *B. fragilis* – *Bacteroides fragilis*

In 41 (63%) of 65 implants removed due to presumed aseptic causes, no bacteria were isolated from SF culture. The corresponding tissue cultures in these implants showed either no bacterial growth in 38 cases or growth that was classified as likely contamination in three cases. In the remaining 24 (37%) implants, 27 bacterial isolates were isolated from SF culture (Table 2 + 3). Eleven (41%) isolates from SF of 11 different implants were classified as indicative of a potential infection according to our study criteria. The remaining 16 (59%) of the 27 isolates were considered likely contaminants (Table 3).

**Table 2:** Details of the eleven implants in the presumed aseptic group, with isolation of bacteria from sonication fluid culture that potentially represent infection

No.	Joint	Type of Implant	Reason for implant removal	Tissue culture^a^	Sonication fluid culture		
					**Organism**	**Colony forming units/ml** ^**b**^	**Time to growth (days)**
1	Shoulder	Arthroplasty	Aseptic failure	*C. acnes* (5/5)	*C. acnes*	20	14
2	Shoulder	Arthroplasty	Aseptic loosening	*C. acnes* (4/5)	*C. acnes*	100	3
3	Shoulder	Arthroplasty	Aseptic failure	*C. acnes* (2/5)	*C. acnes*	60	7
4	Elbow	Arthroplasty	Aseptic loosening	*C. acnes* (2/5) *S. epidermidis* (2/5)	*C. acnes*	> 250	3
5	Hip	Arthroplasty	Fracture	No growth	*S. epidermidis*	> 250	1
6	Elbow	Plate/screw	Pseudoarthrosis	No growth	*B. wiedmannii*	> 250	1
7	Hip	Arthroplasty	Aseptic loosening	No growth	*S. epidermidis*	> 250	14
8	Hip	Arthroplasty	Aseptic loosening	CoNS (1/5)	*S. capitis*	> 250	9
9	Knee	Arthroplasty	Aseptic failure	No growth	*S. warneri*	60	9
10	Shoulder	Arthroplasty	Fracture	No growth	*S. warneri*	200	5
11	Shoulder	Arthroplasty	Aseptic loosening	No growth	*S. epidermidis*	> 250	7
					*C. acnes* ^*c*^	20	7

^a^Numbers in parentheses after the bacterial species give the number of cultures that were positive for the bacteria/total number of cultures taken

^b^The CFU was calculated based on growth in uncentrifuged sonication fluid

^c^ Identified as contaminant based on analyses in the previous study

*C. acnes* – *Cutibacterium acnes*, *S. epidermidis* – *Staphylococcus epidermidis*, *B. wiedmannii* – *Bacillus wiedmannii*, CoNS *–* Coagulase negative *Staphylococcus, S. capitis* – *Staphylococcus capitis*, *S. warneri* – *Staphylococcus warneri*

**Table 3:** Details of the thirteen implants in the presumed aseptic group, with isolation of contaminant bacteria (n=15) on sonication fluid culture

No.	Joint	Type of Implant	Reason for implant removal	Tissue culture	Sonication fluid culture		
					**Organism**	**Colony forming units/mL** ^**a**^	**Time to growth in days**
1	Hip	Arthroplasty	Aseptic loosening	No growth	*C. acnes*	50	6
					*S. capitis*	50	6
2	Shoulder	Arthroplasty	Instability	No growth	*C. acnes*	20	6
3	Knee	Arthroplasty	Aseptic failure	No growth	*C. acnes*	> 250	21
4	Hip	Arthroplasty	Aseptic failure	No growth	*C. acnes*	20	7
5	Shoulder	Arthroplasty	Instability	No growth	*C. acnes*	20	21
6	Shoulder	Arthroplasty	Glenoid attrition	No growth	*C. modestum*	40	7
7	Hip	Arthroplasty	Malposition	No growth	*C. modestum*	> 250	3
8	Elbow	Arthroplasty	Aseptic loosening	No growth	*C. modestum*	160	14
9	Shoulder	Arthroplasty	Instability	No growth	*S. aureus*	10	5
10	Knee	Arthroplasty	Instability	No growth	*S. simulans*	10	2
11	Shoulder	Arthroplasty	Aseptic failure	No growth	*S. epidermidis*	20	21
12	Shoulder	Arthroplasty	Glenoid attrition	No growth	*S. epidermidis*	30	14
13	Hip	Arthroplasty	Aseptic failure	No growth	*S. pasteuri*	20	5
					*C. avidum*	200 (C)	20

^a^ CFU/ml was calculated from growth in uncentrifuged sonication fluid except for *C. avidum* where centrifuged (C) sonication fluid was used

*C. acnes* – *Cutibacterium acnes*; *C. modestum* – *Cutibacterium modestum*; *C. namnetense* – *Cutibacterium namnetense*; *S. aureus* – *Staphylococcus aureus*; CoNS – Coagulase negative Staphylococcus; *S. epidermidis* – *Staphylococcus epidermidis*; *S. warneri* – *Staphylococcus warneri*; *S. pasteuri* – *Staphylococcus pasteuri*; *S. capitis* – *Staphylococcus capitis*; *S. simulans* – *Staphylococcus simulans*

**Table 4:** Reasons for classification of the seventeen bacterial isolates as contaminants

Bacterial Species	Number	Reason for exclusion
*Cutibacterium acnes*	6	Based on combined analyses of culture-dependent and -independent analyses of SF in our previous publication [16].
*Cutibacterium modestum*	3	Initially identified as *C. acnes* by 16S rRNA sequencing and then reassigned as *C. modestum* based on whole genome sequencing. Regarded as contaminants in our previous publication [16].
*Cutibacterium avidum*	1	Growth below CFU/ml cut-off
*Cutibacterium namnetense* ^a^	1	Growth below CFU/ml cut-off
*Staphylococcus aureus*	1	Growth below CFU/ml cut-off
CoNS	5	Growth below CFU/ml cut-off

CFU/ml – Colony forming units/ml of sonication fluid (SF) with > 50 CFU/ml of uncentrifuged SF or > 200 CFU/ml of centrifuged SF used as cut-offs; CoNS – Coagulase negative *Staphylococcus* species

^a^ Isolate is from the confirmed/suspected IAI group

## Discussion

In this study, we applied sonication with SF culture on 77 removed orthopaedic implants, with concomitant tissue biopsies.

We found that for implants in which the surgeon had noted infection as the cause of removal, SF culture helped improve the diagnostic yield in two patients. In the first patient (Table 1, No. 2), who clinically presented with a sinus tract, the causative agent (*C. acnes*) was detected only in SF culture and not in tissue culture. In the second patient (Table 1, No. 11) in whom infection was suspected because of raised inflammatory markers, SF culture confirmed the diagnosis and the causative agent. Similarly, in the presumed aseptic group (Table 2), SF culture improved the diagnostic yield in seven patients, six of whom had no growth on tissue culture, and one that had bacterial growth in only one of the five tissue culture specimens.

The use of SF culture in the diagnosis of IAI is widely used internationally and has been studied since 2007 [14]. Although current literature primarily originates from dedicated infection centres or large tertiary referral centres and results are slightly conflicting, with some suggesting that SF culture is superior to tissue culture [12, 14, 21–23], while others report better or similar performance of tissue culture [24–27]. The reported discordance in literature could be due to lack of standardization in the number of tissue samples collected, variations in the sample processing methods, and differences in the growth conditions and CFU/ml cut-offs for positive sonication fluid cultures used in different laboratories.

We also found that there is a substantial risk for the isolation of contaminants in SF culture, especially in the presumed aseptic group, despite taking adequate precautions in the handling of the implants [16] (Tables 4 and 3). Of the 49 bacterial isolates from SF, more than half were considered contaminants according to the criteria used in the study, and all but one of these were from implants removed due to aseptic causes. *Cutibacterium* species and CoNS accounted for nearly 90% of the contaminants. In comparison, only about a quarter of the bacterial isolates from tissue samples were considered contaminants. A study by Namdari et al. [28] reported a risk of contamination of samples by *C. acnes* and CoNS that originated from the air in the operating room. In implant removal surgeries, samples for tissue culture are collected relatively soon after skin incision and using strict aseptic methods, while prostheses are exposed to the air in the operating room for a longer duration before retrieval since the extraction can be a tedious and long-standing process. Such differences may explain the higher risk of contamination seen in SF culture of extracted prostheses. In addition, contamination in the laboratory during implant processing and incubation is a potential source of contamination [16]. Use of multiple agar plates, prolonged incubation of SF cultures (28 days versus up to 14 days for most tissue cultures) as well as repeated checking of plates for growth during this period could explain the increased contamination rates of SF cultures in this study.

*C. acnes* was the most common bacteria isolated from implants in the aseptic group and the second most common organism in the infection group (Tables 1, 2 and 3). All the *C. acnes* isolates that would represent infection and more than half from the contamination group originated from upper extremity implants (shoulder/elbow). These results correlate with previous studies that have shown that *C. acnes* has a predilection for the upper extremity [7, 29, 30]. Both et al. also reported that *C. acnes* was commonly detected from clavicle plates of seemingly infection-free patients, while being absent in fibular implants [31]. We used additional criteria to determine the significance of *C. acnes* isolates in the study. Interestingly, if the SF culture results were interpreted based on EBJIS cut-off for SF culture, then two *Cutibacterium* isolates considered to represent infection might have been missed. While the guidelines mention that any positive culture in SF be considered as a ‘potential infection’, it also states that low growth of common contaminants like *C. acnes* are less likely to represent infection compared to more virulent bacteria like *S. aureus* [19]. Moreover, *C. acnes* is often the most common bacteria obtained in culture of implants without prior suspicion of infection [30, 32–36] though the clinical impact of these unexpected positive cultures in the upper extremity is currently unclear [36, 37]. More research into this area is needed. Similarly, three of the *Cutibacterium* isolates that were considered contaminants in this study would have fallen under the ‘infection confirmed’ category in the EBJIS guidelines [19]. Accordingly, interpretation of positive *Cutibacterium* species cultures must always be with caution.

The study has several limitations. Due to the exploratory nature of the study, only a limited number of implants were included. The decision to send tissue cultures was dependent on the operating surgeon, further limiting the number of implants included in the study. Another main drawback of the study was that classification of implants into the presumed aseptic group, or the infected group was based on what was written in the patients’ charts by the operating surgeon and not based any internationally recognized criteria like the EBJIS guidelines. This was due to the unavailability of all the required data in the patients’ charts. Finally, we did not perform a clinical follow-up. As such, the clinical relevance of *C. acnes* in the presumed aseptic group including the isolates considered non-contaminants is still unclear.

## Conclusion

Sonication of implants irrespective of reason for removal appears to be of added value in the detection of the causative organisms. However, SF cultures should be interpreted with care especially when *Cutibacterium* species are isolated, irrespective of the amount of growth.

## Data Availability

Not relevant.

## Data Availability

The datasets used during the current study are available from the corresponding author on reasonable request.
